# Advances in Studies on Microbiota Involved in Nitrogen Removal Processes and Their Applications in Wastewater Treatment

**DOI:** 10.3389/fmicb.2021.746293

**Published:** 2021-09-28

**Authors:** Wenning Mai, Jiamin Chen, Hai Liu, Jiawei Liang, Jinfeng Tang, Yongjun Wei

**Affiliations:** ^1^ School of Ecology and Environment, Zhengzhou University, Zhengzhou, China; ^2^ College of Public Health, Zhengzhou University, Zhengzhou, China; ^3^ Laboratory of Synthetic Biology, Zhengzhou University, Zhengzhou, China; ^4^ Henan Public Security Bureau, Zhengzhou, China; ^5^ Key Laboratory for Water Quality and Conservation of Pearl River Delta, Ministry of Education, School of Environmental Science and Engineering, Linköping University – Guangzhou University Research Center on Urban Sustainable Development, Guangzhou University, Guangzhou, China; ^6^ Key Laboratory of Advanced Drug Preparation Technologies, Ministry of Education, School of Pharmaceutical Sciences, Zhengzhou University, Zhengzhou, China

**Keywords:** nitrogen pollution removal, nitrifying bacteria, denitrifying bacteria, anammox, microbiome, wastewater

## Abstract

The discharge of excess nitrogenous pollutants in rivers or other water bodies often leads to serious ecological problems and results in the collapse of aquatic ecosystems. Nitrogenous pollutants are often derived from the inefficient treatment of industrial wastewater. The biological treatment of industrial wastewater for the removal of nitrogen pollution is a green and efficient strategy. In the initial stage of the nitrogen removal process, the nitrogenous pollutants are converted to ammonia. Traditionally, nitrification and denitrification processes have been used for nitrogen removal in industrial wastewater; while currently, more efficient processes, such as simultaneous nitrification-denitrification, partial nitrification-anammox, and partial denitrification-anammox processes, are used. The microorganisms participating in nitrogen pollutant removal processes are diverse, but information about them is limited. In this review, we summarize the microbiota participating in nitrogen removal processes, their pathways, and associated functional genes. We have also discussed the design of efficient industrial wastewater treatment processes for the removal of nitrogenous pollutants and the application of microbiome engineering technology and synthetic biology strategies in the modulation of the nitrogen removal process. This review thus provides insights that would help in improving the efficiency of nitrogen pollutant removal from industrial wastewater.

## Introduction

Industrial development improves our life quality; nevertheless, the industries, such as those producing paper and pharmaceutical products, generate large amounts of industrial wastewater (Liang et al., 2021; Singh et al., 2021). Nitrogen is one of the main industrial wastewater pollutants (Sun et al., 2021), the spread of which pollutes the environment (Chen et al., 2018), damages the ecosystem, and affects human health (Liu et al., 2021). Nitrogenous pollutants in wastewater mainly comprise inorganic nitrogen and organic nitrogen (Odedishemi Ajibade et al., 2021). The organic nitrogen pollutants can be catalyzed by microorganisms to form inorganic pollutants (Wei et al., 2015). Thus, the primary task of wastewater treatment is the removal of inorganic nitrogen. Therefore, developing green and sustainable strategies to remove inorganic nitrogen pollutants is of great interest (Deng et al., 2021).

Both physicochemical and biological methods are used for removing nitrogenous pollutants in wastewater. The physicochemical methods include stripping, wet oxidation technology, electrochemical technology (Monfet et al., 2018), ion exchange, and adsorption methods (Mook et al., 2012). While physicochemical methods require higher capital and generate solid wastes as secondary contamination, biological methods are mainly used for the efficient removal of nitrogen pollutants (Monfet et al., 2018; Wang et al., 2020c; Chen et al., 2021b). Inorganic nitrogen pollutants are mainly available in the form of ammonia nitrogen (NH_4_^+^-N), nitrite nitrogen (NO_2_^−^-N), and nitrate nitrogen (NO_3_^−^-N). Biological removal of these nitrogen pollutants in wastewater treatment plants mainly involves the process of ammonification, nitrification, denitrification, and anammox processes (Guo et al., 2020; Liu et al., 2020). These nitrogen removal processes convert nitrogen pollutants to several different oxidation states, and each process needs special running parameters (Rahimi et al., 2020). In each process, different microorganisms function and varying metabolic reactions are involved, and the efficiency of each nitrogen removal process is divergent (Zhang et al., 2021b). Hence, understanding the biological removal processes at species and molecular level is essential for the development of efficient nitrogen pollution removal strategies.

In this review, we aim to summarize the nitrogen removal processes and their microbiota used for the removal of nitrogen pollutants, their functional genes, metabolic pathways, and associated mechanisms. The application and optimization of nitrogen pollution removal process are systematically described, and their operating effectiveness is compared. Based on current nitrogen removal processes, we also discuss and propose the future application of these functional microorganisms and their engineering for industrial wastewater treatment *via* microbiota engineering and synthetic biology strategies.

## Biological Denitrification Process for Nitrogen Pollutant Removal

The biological nitrogen pollutant removal process mainly involves partial nitrification (PN), nitrification, denitrification, and anammox (Supplementary Table S1 and Supplementary information). The microbial processes and their associated genes involved in nitrogen removal have been identified in previous studies (Supplementary Figure S1; Wang et al., 2014; Rahman et al., 2018; Li et al., 2021b). The nitrification process converts ammonia nitrogen into nitrate nitrogen and involves ammonia-oxidizing bacteria (AOB) and nitrite-oxidizing bacteria (NOB). AOB and NOB are autotrophic Gram-negative aerobic bacteria that use the energy released in the nitrification process for growth. First, ammonia nitrogen is transformed into nitrite nitrogen by AOB (Mehrani et al., 2020) through the PN process (Wang et al., 2020a), a complex biochemical process that involves electron transfer, and generates energy and diverse intermediates (Xia et al., 2019; Ren et al., 2020; Qian et al., 2021). The process initiates by oxidation of NH_4_^+^-N to hydroxylamine (NH_2_OH) by ammonia monooxygenase, which is then oxidized to nitrite nitrogen by hydroxylamine oxidoreductase. The nitrite nitrogen is further transformed into nitrate nitrogen by nitrite oxidoreductase of NOB (Staley et al., 2018).

Denitrification is an important step of the biological nitrogen cycle (Zhang et al., 2019); it involves several enzymes and generates various intermediate metabolites (Ren et al., 2020). Four key enzymes of nitrate reductase, nitrite reductase, nitric oxide reductase, and nitrous oxide reductase catalyze the transformation of the nitrate to nitrogen gas (Ding et al., 2019). Most denitrifying bacteria, being heterotrophic facultative anaerobes, carry out the reaction under anaerobic conditions in two steps using nitrate as an electron acceptor and organic matter (organic carbon) as electron donor (Semedo et al., 2018).

## The Traditional Biological Nitrogen Removal Process and Simultaneous Nitrification-Denitrification Process

**Figure 1:**
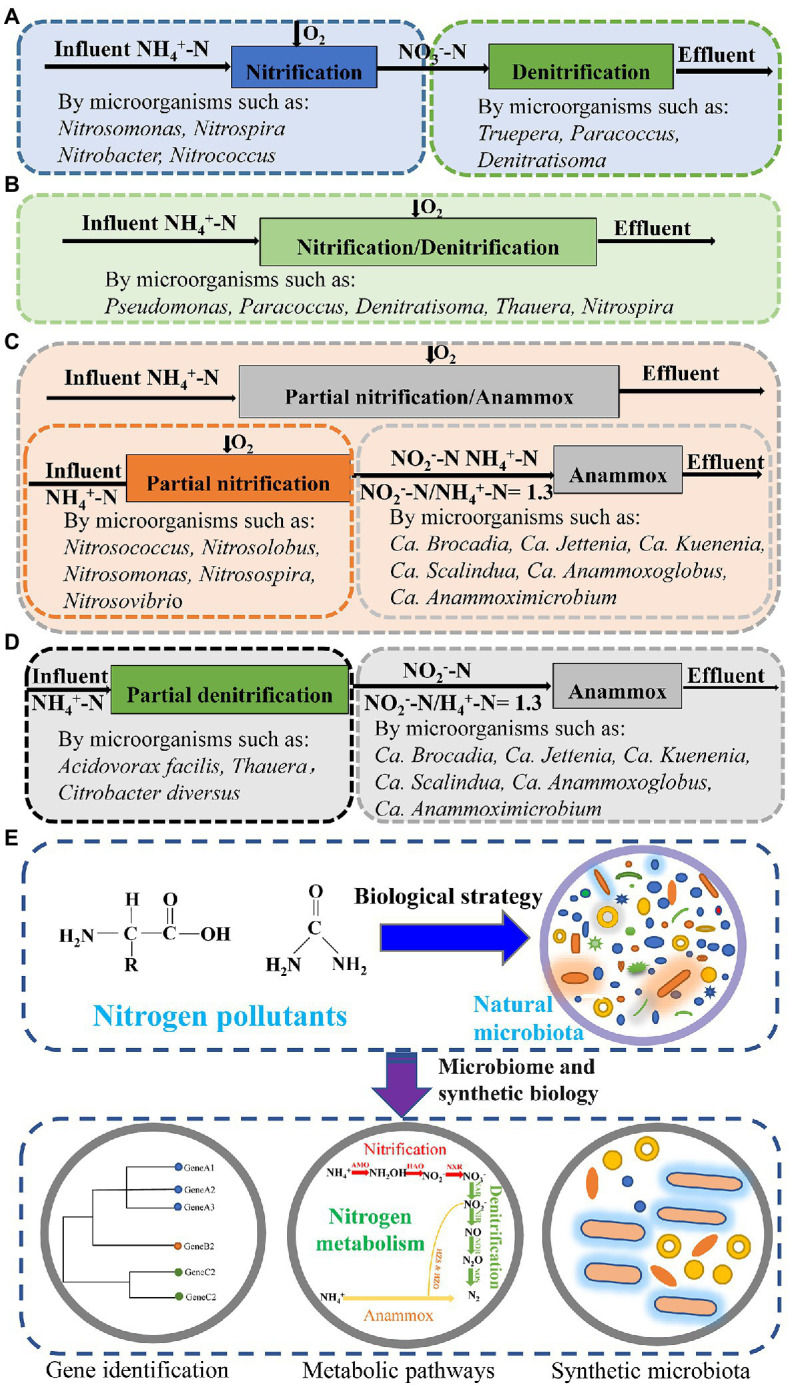
Biological nitrogen removal processes and the microorganisms involved in these processes. **(A)** Traditional nitrification and denitrification processes. **(B)** Simultaneous nitrification-denitrification process. **(C)** Partial nitrification-anammox process. **(D)** Partial denitrification-anammox process. **(E)** Microbiome and synthetic biology strategy used for nitrogen pollutant removal process. The natural microbiota is used for nitrogen pollutant removal; with the help of microbiome and synthetic biology strategy, new nitrogen removal strains can be isolated and engineered strains can be constructed; and these strains can be engineered for synthetic microbiota with efficient nitrogen removal ability.

The traditional biological nitrogen removal (BNR) process involves sequential, full-scale nitrification and denitrification reactions to transform ammonia nitrogen into nitrogen gas as: NH_4_^+^→NO_2_^−^→NO_3_^−^→NO_2_^−^→N_2_. This process has been applied for effectively removing nitrogen pollutants from the wastewater (Kornaros et al., 2010; Chen et al., 2021c; Zhang et al., 2021a; Figure 1A). Based on the BNR process, simultaneous nitrification-denitrification (SND) process has been developed, wherein, the nitrification and denitrification reactions occur synchronously in the same reactor and convert ammonia nitrogen into nitrogen gas (Wang et al., 2006).

Compared with the traditional BNR process, the SND process reduces the investment in equipment and space occupation and is thus a cost-effective process for nitrogen pollutant removal from industrial wastewater (Supplementary Table S2; Xiang et al., 2020). The microorganisms involved in the SND process are mainly nitrifying bacteria and aerobic denitrifying bacteria (Figure 1B). The primary factors affecting the nitrogen removal efficiency include the carbon to nitrogen ratio (COD/N), dissolved oxygen (DO) concentration, sludge concentration, and pH (Chang et al., 2019). Especially, the simultaneous nitrification-denitrification process requires the simultaneous presence of aerobic and anaerobic environments within the same reactor; hence, the DO concentration directly affects the denitrification rate and efficiency (Wang et al., 2018). Moreover, the SND process had been applied for the removal of phosphorus pollutants from municipal wastewater, showing the SND process is feasible in phosphorus removal (Salehi et al., 2019).

Due to the requirements of proper DO and COD/N, the establishment of SND process and sustaining SND process at high efficiency and a stable state for industrial wastewater treatment is difficult (Lai et al., 2020). Some novel microorganisms, including aerobic denitrifying bacteria, low DO nitrifying bacteria, heterotrophic denitrifying bacteria, and some autotrophic denitrifying bacteria, have been identified and used to improve the efficiency and robustness of SND process (Wang et al., 2017; Carneiro Fidélis Silva et al., 2019). Moreover, optimization carbon-to-nitrogen ratio, DO concentration, carrier materials, and other strategies have been used for SND startup and stable running (Dobbeleers et al., 2017; Iannacone et al., 2019; Salcedo Moyano et al., 2021). However, the denitrification process under aerobic conditions is rarely reported, and little information about the SND microbiota is available (Liu et al., 2019; Li et al., 2021a). In the future, giving insights into the SND process and optimizing SND startup, including the design of proper wastewater treatment plant, dynamic microbiota of the running bioreactors, and recovering the association between functional microbiota and running performance, are necessary for industrial-scale nitrogen wastewater treatment with SND process.

## Anaerobic Ammonium Oxidation Process for Nitrogen Pollutant Removal

In 1995, anaerobic ammonium oxidation (Anammox)—a revolutionary process—was identified during a denitrification process for wastewater treatment (Mulder et al., 1995). This discovery provides an understanding of the available nitrogen processing in nature and is a novel applicable process for the removal of nitrogen pollutants (Speth et al., 2016). Anammox process can efficiently remove nitrogen pollutants in the wastewater containing high levels of ammonia nitrogen and low levels of organic pollutants. This process is being applied these days in hundreds of large-scale wastewater treatment plants (Ali and Okabe, 2015) and can potentially treat low-strength nitrogen wastewater by optimizing reactor types and operation parameters (Li et al., 2021d).

## The Anammox Process for Nitrogen Removal

In the anammox process, anammox bacteria directly convert ammonia nitrogen and nitrite nitrogen into nitrogen gas, using ammonia nitrogen as the electron donor and nitrite nitrogen as the electron acceptor in anaerobic environments (Chen et al., 2021a). First, NO_2_^−^-N is reduced to NO, which is used as the electron acceptor of NH_4_^+^-N to produce N_2_H_4_. N_2_H_4_ is further oxidized to form N_2_ (van de Graaf et al., 1997). The anammox process is low cost because no energy input is needed (Xu et al., 2020). The bacteria involved in the anammox process are different from those in the traditional BNR process (Supplementary Table S2; Zhu et al., 2008; Wen et al., 2020).

The anammox process requires NO_2_^−^ as an electron acceptor, but the wastewater often contains NH_4_^+^ and no NO_2_^−^. This NO_2_^−^ can be provided by the PN process for initiation and continuation of the anammox process (Chen et al., 2020). The partial nitrification-anammox (PN/A) process is a short biological denitrification method that can achieve high efficiency of denitrification at a proper temperature, DO concentration, hydraulic retention time, and pH (Val Del Rio et al., 2019; Zhang et al., 2019) with the help of AOB and anammox bacteria (Lv et al., 2011; Figure 1C). This process can efficiently remove nitrogen pollutants without adding organic carbon sources and controlling wastewater COD concentration (Sheng et al., 2020).

The PN/A process can save about 50% oxygen with low sludge generation, and no release of CO_2_ into the air (Huang et al., 2020). According to the available estimates, the PN/A process can save more than 90% of the operating cost (Zhao et al., 2021). However, the low growth rate of anammox bacteria, the low robustness of anammox bacteria to environmental changes, and the nitrogen removal rate limited the application of anammox for nitrogen pollutant removal (Weralupitiya et al., 2021; Wang et al., 2021c). The quorum sensing strategy had been proposed for improving functions of the PN/A process, which might enhance nitrogen removal efficiency through PN/A process in the future (Zhao et al., 2021).

## The Partial Denitrification Process Used for Nitrogen Removal

Partial denitrification (PDN) stops the reduction of Nitrite nitrogen to nitrogen and is considered to be an alternative process for providing nitrite to anammox bacteria (Fu et al., 2019; Cui et al., 2020). By treating wastewater with high-level nitrate nitrogen and low-level ammonia nitrogen, the PDN-anammox (PDN/A) process can reduce organic carbon source input and generate less sludge (Zhang et al., 2020). The microorganisms mainly functioned in the PDN process are partial denitrifying bacteria and anammox bacteria, including *Acidovorax facilis*, *Citrobacter diversus*, and some *Thauera* species (Figure 1D; Wang et al., 2020d).

AOB and anammox bacteria (AnAOB) are the primary functional microorganisms in the PN process and anaerobic ammonia oxidation, and they are also essential for autotrophic denitrification (Wu et al., 2019). However, the PN/A process can produce more than 11% nitrate nitrogen using one-stage or two-stage PN/A processes, which needs to be processed further (Li et al., 2020b). The combination of denitrification PN, and anammox processes (DN-PN/A) in a self-circulating integrated plant is a promising and efficient process to remove nitrogen pollutants from wastewater (Yan et al., 2020). The primary microorganisms involved in the process are AOB, AnAOB, and denitrifying bacteria (Du et al., 2021), and the reactions involved in the DN-PN/A process are as:

$$\begin{array}{l} \mathrm{Partial nitrification}:\\ {\mathrm{NH}}_{4}{}^{+}+1.5\;O_{2}\to {\mathrm{NO}}_{2}{}^{-}+{\mathrm{2H}}^{+}+H_{2}O \end{array}$$ (1)

$$\begin{array}{l} \mathrm{Anammox}:\\ {\mathrm{NH}}_{4}{}^{+}+1.32\;{\mathrm{NO}}_{2}{}^{-}\to 1.02N_{2}+0.26{\mathrm{NO}}_{3}{}^{-}+2.03H_{2}O \end{array}$$ (2)

$$\begin{array}{l} \mathrm{Denitrification reaction}:\\ {\mathrm{8NO}}_{3}{}^{-}+{\mathrm{5CH}}_{3}\mathrm{COOH}\to 10{\mathrm{CO}}_{2}+4N_{2}+{\mathrm{8OH}}^{-}+6H_{2}O \end{array}$$ (3)

In principle, the DN-PN/A process can remove 100% of ammonia nitrogen, but it is difficult to create a balance between the growth of heterotrophic microorganisms and autotrophic microorganisms (AOB, AnAOB, and other microorganisms) in one integrated reactor (Ma et al., 2020). Thus, research needs to be conducted to develop or engineer optimized DN-PN/A microbiota (Jiang et al., 2021).

## Industrial Application of BNR for Wastewater Treatment

The traditional biological denitrification process is based on three reactions, including ammonification, nitrification, and denitrification, and the associated microorganisms can be accumulated as activated sludge (Supplementary Figure S2). The ammoniation reaction takes place in the aeration tank and can remove organic carbon and transfer organic nitrogen to NH_4_^+^-N (Supplementary Table S1). After precipitation, the effluent from the ammoniation process enters the nitrification tank where NH_4_^+^-N is converted to NO_3_^−^-N. The nitrification reaction requires an acid to decrease the pH of the reactor. The NO_3_^−^-N is reduced to N_2_ in the denitrification process, which requires organic carbon sources, such as methanol and glucose. In practice, original wastewater containing organic carbon is mixed with the nitrification effluent (Falås et al., 2016).

In addition to the described processes, the anaerobic-aerobic process (A/O) or recurring denitrification process is also used for removing nitrogen pollutants. The A/O process can efficiently use original organic compounds in wastewater, reduce air input, and in the process, the intermediate tank and reflux system are removed (Zhang et al., 2013). The A/O process significantly reduces construction and operation costs. Based on the A/O process, the anaerobic/anoxic/aerobic (A^2^/O) process is optimized to carry out the denitrification and dephosphorization processes, which can be synchronously in one reactor, and simultaneously remove the phosphorus, showing that traditional biological wastewater treatment strategy is efficient and cost-friendly (Park et al., 2021).

To conduct operations for nitrogen removal, the microbiota of the nitrogen removal processes is examined. *Nitrospira*, *Thauera*, *Dechloromonas*, and *Ignavibacterium* are the most abundant microbial genera in the A^2^/O sludge (Kim et al., 2013; Xiang et al., 2021). Further, *Nitrosomonas*, *Nitrospira*, and *Nitrobacter* have been identified as the key taxa for nitrite oxidation (Wang et al., 2019; Li et al., 2020a; Feng et al., 2021; Zhou et al., 2021), and *Truepera, Paracoccus*, and *Denitratisoma* were found to primarily carry out denitrification (Wang et al., 2019; Deng et al., 2020; Li et al., 2020a; Wang et al., 2020b). Recently, the autotrophic nitrogen removal systems, including PN, anammox, and the PN/A processes in two bioreactors or in a single bioreactor, were used as cost-effective ways to treat NH_4_^+^ rich wastewater (Dehestaniathar et al., 2021).

The anammox process for industrial wastewater treatment was developed in China more than a decade ago (Ni et al., 2010). For synthetic wastewater treatment, the primary functional anammox microbes were identified to be *Nitrosomonas*, *Stuttgartiensis*, and *Candidatus Kuenenia* (Zhang et al., 2013). The anammox process has also been used for the treatment of vitamin B_2_ production wastewater, and *Ca. Kuenenia* and *Nanaocystis* were found to be the main functional microorganisms (Table 1; Mai et al., 2020). Besides, new anammox bacterial species and sulfate-dependent anammox bacteria, such as *Anammoxoglobus sulfate* (Liu et al., 2008) and *Bacillus benzoevorans* (Cai et al., 2010), were found to assist in removing ammonium and sulfate simultaneously during wastewater treatment (Nie et al., 2021). Currently, with the aid of molecular techniques, at least five genera of anammox bacterial have been identified, including *Ca. Brocadia* (Kartal et al., 2008), *Ca. Kuenenia* (Schmid et al., 2000), *Ca. Scalindua* (Ali et al., 2020), *Ca. Anammoxoglobus* (Kartal et al., 2007), and *Ca. Jettenia asiatica* (Ali et al., 2013). However, no pure culture of these anammox has been obtained yet. In the future, culturomics may contribute to the isolation of anammox bacteria and help unravel nitrogen metabolic pathways of anammox (Lagier et al., 2018).

**Table 1:** Biological nitrogen removal processes for different wastewater types.

Wastewater types	Main process	Nitrogen removal microorganisms in the microbiota	References
Domestic wastewater	anaerobic/anoxic/aerobic (A^2^/O)	*Dechloromonas; Nitrospira; Arcobacter; Dokdonella*	Xiang et al., 2021
Campus wastewater	Synchronous nitration denitrification (SND)	*Nitrospira; Thermomicrobia; Denitratisoma; Rhodocyclaceae*	Xiang et al., 2020
Synthetic wastewater	Anammox	*Candidatus* Scalindua; *Actinomarinales*	Zhang et al., 2013
Sewage	Partial denitrification-anammox (PDN/A)	*Thauera; Candidatus Brocadia*	Wang et al., 2020b
Landfill leachate	Partial nitrification-denitrification (PND)	*Nitrosomonas; Nitrospira; Ottowia; Pseudomonas; Thermomonas; Thiobacillus; Paracoccus; Thauera; Arenimonas*	Li et al., 2020a
Mature landfill leachate	Simultaneous partial nitrification, anammox and denitrification (SNAD)	*Nitrosomonas; Chloroflexi; Ignavibacteria; Candidatus Brocadia; Candidatus Jettenia*	Wang et al., 2019
Municipal wastewater	Partial nitrification-simultaneous anammox and denitrification (PN-SAD)	*Limnobacter; Ignavibacter; Thauera; Denitration; Candidatus Brocadia*	Deng et al., 2020
Piggery wastewater	Heterotrophic nitrification-anammox	*Candidatus Kuenenia; Planctomyces; Pirellula; Hyphomicrobium; Rhodobacter; Ignavibacterium*	Zhou et al., 2021
Vitamin B_2_ production wastewater	Anammox	*Candidatus Kuenenia; Nanaocystis*	Mai et al., 2020
Domestic sewage	Anaerobic/Aerobic/Anoxic/Aerobic process (AOAO)	*Dechloromonas; Candidatus Competibacter*; *Nitrospira; Nitrosomonas*	Feng et al., 2021

## The Application of Microbiome and Synthetic Biology for Nitrogen Removal

High-throughput sequencing techniques, metagenomics, and other microbiome strategies are being applied to analyze microbiota with the ability to remove nitrogen pollutants (Xiang et al., 2021). There is a great diversity in the dominant microorganisms functioned in different nitrogen pollutant removal processes. Nevertheless, most microorganisms are assigned to the phyla of Proteobacteria, Bacteroidetes, Nitrospirae, and Chloroflexiphyla (Table 1), and some bacteria in the ammonification, nitrification, and denitrification processes have already been isolated (Table 1). Although several anammox bacteria have been identified using molecular techniques, no pure culture of the anammox bacteria has yet been obtained (Table 1; Zhang and Okabe, 2020).

In the future, microbiome strategies can be used to discover anammox genomes and the functional genes in the PN/A microbiota and other microbiota. Based on metabolic information inferred from the microbiome data, a proper medium can be designed for the isolation or enrichment of anammox bacteria (Wei et al., 2020). Besides, the functional genes and pathways discovered in the microorganisms that can remove nitrogen pollutants can be expressed in the model organisms, such as *Escherichia coli* (Wang et al., 2021a), *Clostridium perfringens* (Wang et al., 2011), *Klebsiella pneumoniae* (Wang et al., 2021b), and others (Wang et al., 2020c), to build genetically engineered strains for nitrogen pollutant removal (Figure 1E). These isolated strains, engineered strains, and enriched microbiota can be used for the construction of a series of synthetic microbiota with nitrogen removal ability, as well as those that can accomplish different nitrogen removal processes (Jiang et al., 2021; Li et al., 2021c). Based on the nitrogen pollutant types and concentration, proper synthetic microbiota can be selected and developed for nitrogen pollutant removal (Figure 1E).

## Perspectives

In this review, current biological denitrification processes and associated functional microorganisms have been summarized. The advantages and limitations of current mainstream denitrification processes in wastewater treatment have also been reviewed, and PN/A, PDN/A, DN-PN/A, and other anammox processes might be the main nitrogen removal strategies in the next few years. In order to enhance nitrogen removal efficiency, proposing novel integrated process for nitrogen removal and giving insight into the molecular mechanisms of each nitrogen removal process are essential for nitrogen pollutant removal in the industrial-scale wastewater. Moreover, some primary nitrogen pollutant removal bacteria have not yet been cultured in the laboratory, and microbiome should be implemented for the recovery of microorganisms functioned in the nitrogen pollutant removal process. In the future, synthetic biology strategies would help construct/synthesize microbiota for the efficient treatment of nitrogen pollutants in wastewater based on the nitrogen removal isolates and engineered microbial strains.

## Author Contributions

YW conceived the study. JC, YW, WM, JT, HL, and JL drafted the manuscript. JC and YW prepared the figures. HL and JT revised the manuscript. All the authors read, revised, and approved the manuscript.

## Funding

This work was supported by the National Natural Science Foundation of China (no. 32111530179), and the Science and Technology Program of Guangzhou, China (no. 202102010401).

## Conflict of Interest

The authors declare that the research was conducted in the absence of any commercial or financial relationships that could be construed as a potential conflict of interest.

## Publisher’s Note

All claims expressed in this article are solely those of the authors and do not necessarily represent those of their affiliated organizations, or those of the publisher, the editors and the reviewers. Any product that may be evaluated in this article, or claim that may be made by its manufacturer, is not guaranteed or endorsed by the publisher.
