# Supplementary material for: Advances in Studies on Microbiota Involved in Nitrogen Removal Processes and Their Applications in Wastewater Treatment
Source: Front Microbiol. 2021 Sep 28;12:746293. doi: 10.3389/fmicb.2021.746293 (PMC8560000; doi:10.3389/fmicb.2021.746293)
Supplement: Supplementary file 1 [file Data_Sheet_1.doc]

**Supplementary information**

**Advances in studies on microbiota involved in nitrogen removal processes and their applications in wastewater treatment**

Wenning Mai^1,2#^, Jiamin Chen^1,3#^, Hai Liu^4^, Jiawei Liang^2^, Jinfeng Tang^5^, Yongjun Wei^3,6*^

^1^School of Ecology and Environment, Zhengzhou University, Zhengzhou, China

^2^College of Public Health, Zhengzhou University, Zhengzhou, China

^3^Laboratory of Synthetic Biology, Zhengzhou University, Zhengzhou, China

^4^Henan Public Security Bureau, Zhengzhou, China

^5^Key Laboratory for Water Quality and Conservation of Pearl River Delta, Ministry of Education, School of Environmental Science and Engineering, Linköping University – Guangzhou University Research Center on Urban Sustainable Development, Guangzhou University, Guangzhou China

^6^Key Laboratory of Advanced Drug Preparation Technologies, Ministry of Education, School of Pharmaceutical Sciences, Zhengzhou University, Zhengzhou, China

^#^These two authors contributed equally to this work.

**^*^**Corresponding author

**Yongjun Wei**

E-mail: [yongjunwei@zzu.edu.cn](mailto:yongjunwei@zzu.edu.cn)

TEL:  +86 (0)371 67781908

The biological nitrogen pollutant removal process mainly composed with the reactions of partial nitrification, nitrification, denitrification, and anammox. The chemical reactions are listed as below:

**Partial nitrification process:**

NH_3_ + O_2_+ 2H^+^ +2e^-^→NH_2_OH + H_2_O (1)

NH_2_OH + H_2_O→HNO_2_ ^+^ 4H^+^ + 4e^-^ (2)

Partial nitrification: NH_4_^+^ + 1.5O_2_ →NO_2_^-^ + 2H^+^ + H_2_O (3)

**Nitrification process:**

Partial nitrification: NH_4_^+^ + 1.5O_2_ →NO_2_^-^ + 2H^+^ + H_2_O (1)

Nitrite oxidation reaction: NO_2_^-^ + 0.5O_2_ → NO_3_^-^ (2)

Nitrification: NH_4_^+^ + 2O_2_→NO_3_^-^ + H_2_O+ 2H^+^ (3)

**Denitrification process:**

6NO_3_^-^ + 2CH_3_OH → 6NO_2_^-^ + 2CO_2_ + 4H_2_O (1)

6NO_2_^-^ + 3CH_3_OH→ 3N_2_ + 3CO_2_ + 3H_2_O + 6OH^-^  (2)

Denitrification process：8NO_3_^-^ + 5CH_3_COOH → 4N_2_ + 10CO_2_ + 6H_2_O + 8OH^-^  (3)

**Anammox process:**

NO_2_^-^ + 2H^+^ + e^-^ = NO + H_2_O (1)

NO + NH_4_^+^ + 2H^+^ + 3e^-^= N_2_H_4_ + H_2_O (2)

N_2_H_4_ = N_2_ + 4H^+^ + 4e^-^  (3)

Anammox: NH_4_^+^ + NO_2_^-^ = N_2_ + 2H_2_O (4)

**Supplementary Tables and Figures**

**Supplementary Tables**

**Table S1** Biological reactions in nitrogen recycling.

**Table S2** Summary of anammox and traditional biological nitrogen removal processes.

**Supplementary Figures**

**Fig S1** Biological nitrogen pollutant removal process and functional genes. AMO, ammonium monooxygenase of ammonia oxidizing bacteria; HAO, hydroxylamine oxidase of ammonia oxidizing bacteria; NXR, nitrite oxidoreductase of nitrite oxidizing bacteria; NAR, nitrate reductase of denitrification; NIR, nitrite reductase of denitrification; NOR, nitric oxide reductase of denitrification; NOS, nitrous oxide reductase of denitrification; HZS, hydrazine synthase of anammox; HZO, hydrazine oxidase of anammox. The amoA and hao genes are the key functional genes involved in partial nitrification. The nxrB gene is the main factor of nitrate nitrogen change. The narG gene is the key functional gene involved in partial denitrification. The nirS/nirK, norB, and nosZ genes are the key functional genes involved in denitrification. The hzsB and hzo genes are the key functional genes involved in anammox.

**Fig S2** The traditional biological denitrification process of activated sludge.

**Table S1** Biological reactions of nitrogen recycling in nature.

| Process | Reaction | Bacteria involved |
| --- | --- | --- |
| Ammonification | Organic nitrogen → NH_4_^+^-N | Amino acid degrading bacteria |
| Partial nitrification | NH_4_^+^-N → NO_2_^-^-N | Ammonia oxidizing bacteria |
| Nitrification | NO_2_^-^-N → NO_3_^-^-N | Nitrite oxidizing bacteria |
| Denitrification | NO_3_^-^-N → NO_2_^-^-N and N_2_ | Denitrification bacteria |
| Anammox | NO_2_^-^-N + NH_4_^+^-N → N_2_ | Anammox |

**Table S2** Summary of anammox and traditional biological nitrogen removal processes.

| Process | Microorganisms | Nitrogen load rate (kg N/m^3^/d) | Oxygen requirements | Organic carbon | TN removal rate (%) |
| --- | --- | --- | --- | --- | --- |
| BNR | Autotrophic+  Heterotrophic | 2-8 | High | Yes | 100 |
| SND | Autotrophic+  Heterotrophic | 1-3.5 | Low | Yes | 100 |
| Anammox | Autotrophic | 5.1 | None | No | 84 |
| PN/A | Autotrophic | 1.2-8.9 | Low | No | 89 |
| DN-PN/A | Autotrophic+  Heterotrophic | Unknown | None | Yes | 100 |


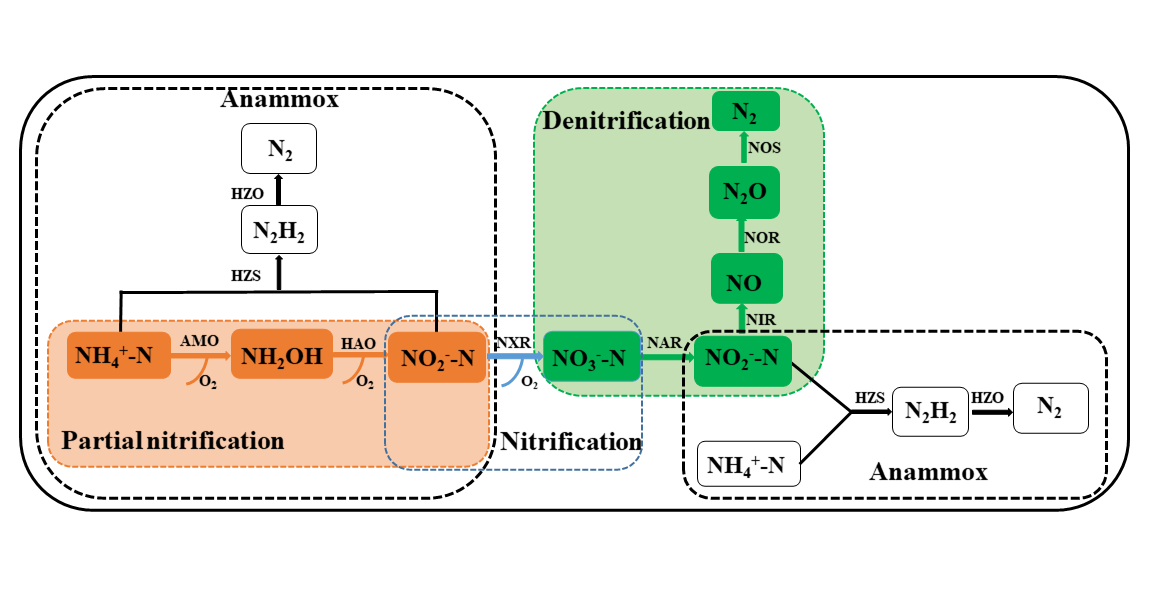


**Fig S1** Biological nitrogen pollutant removal process and functional genes. AMO, ammonium monooxygenase of ammonia oxidizing bacteria; HAO, hydroxylamine oxidase of ammonia oxidizing bacteria; NXR, nitrite oxidoreductase of nitrite oxidizing bacteria; NAR, nitrate reductase of denitrification; NIR, nitrite reductase of denitrification; NOR, nitric oxide reductase of denitrification; NOS, nitrous oxide reductase of denitrification; HZS, hydrazine synthase of anammox; HZO, hydrazine oxidase of anammox. The amoA and hao genes are the key functional genes involved in partial nitrification. The nxrB gene is the main factor of nitrate nitrogen change. The narG gene is the key functional gene involved in partial denitrification. The nirS/nirK, norB, and nosZ genes are the key functional genes involved in denitrification. The hzsB and hzo genes are the key functional genes involved in anammox.


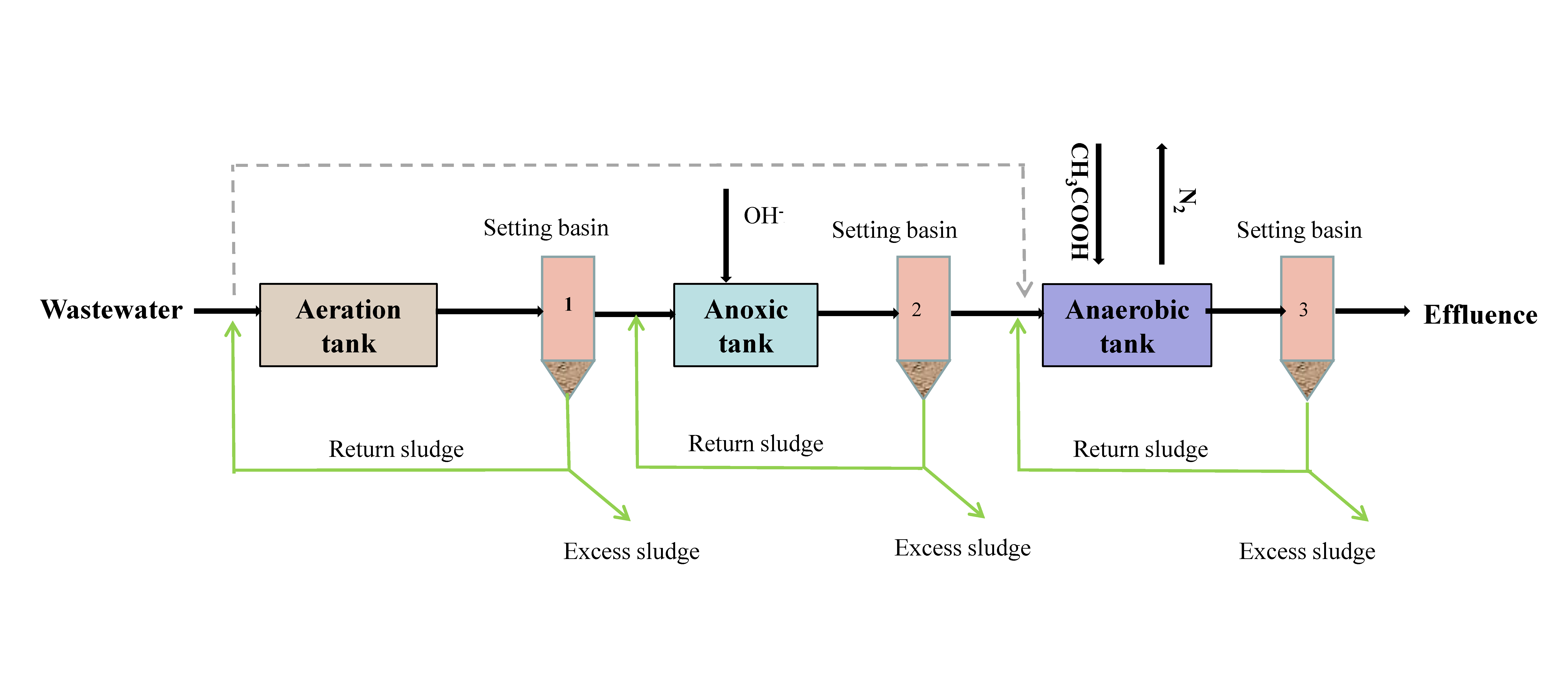


**Fig S2** The traditional biological denitrification process of activated sludge.
